# Toward a framework for understanding translation and interpreting teacher role identity

**DOI:** 10.3389/fpsyg.2022.980196

**Published:** 2022-09-14

**Authors:** Bacui Chen, Jing Huang

**Affiliations:** ^1^School of Foreign Studies, Lingnan Normal University, Zhanjiang, China; ^2^Department of English Language Education, The Education University of Hong Kong, Hong Kong, Hong Kong SAR, China

**Keywords:** translation and interpreting teacher, role identity, trainer/educator, researcher, practitioner, professional development

## Abstract

The purpose of this study was to present a translation and interpreting (T&I) teacher role identity (TITRI) framework for investigating how T&I teachers in China develop their role identities. There is a vast literature on language teacher identity in higher education compared to a paucity of literature on the development of T&I teacher identity. Developing a strong T&I teacher identity in the context of Chinese universities is challenging as teachers combine sub-roles of trainers/educators, researchers, and practitioners, and the context is more supportive of constructing a researcher role than trainer/educator and practitioner roles. A strong teacher identity, on the other hand, is vital since it enhances teachers’ willingness to engage in professional development. This study proposes a framework based on the Dynamic Systems Model of Role Identity (DSMRI) that describes how three sub-roles interact and contribute to overall T&I teacher identity. This work contributes to the scant literature on T&I teacher identity research, shedding light on how different role identities may interact throughout the professional careers of a teacher. Additionally, the framework may also foster an awareness of the impact of higher education on a teacher and, as a result, offer implications for universities in China to encourage the development of teacher identity.

## Introduction

Being a translation and interpreting (T&I hereafter) teacher is demanding as the teacher is expected to be good at training/educating, research, and practice at the same time (Tao, 2019; Kang and Shi, 2020). For one thing, T&I teachers are obliged to carry out a large number of tasks linked to teaching, including both training and education in the T&I degree program (Tan, 2008). For another, because research achievement is heavily weighted in the promotion and incentive scheme in China’s higher education sector (Shu et al., 2020), T&I teachers are under pressure to conduct research in the hope of publishing in high-impact journals, obtaining high-ranking research funding, and receiving government rewards (Long and Huang, 2017). The above two obligations are in line with other language teachers in Chinese contexts, who are mainly non-native speakers (NNS). Furthermore, since the T&I profession is practice-oriented, they are supposed to be actively involved in T&I practice (Kelly, 2008) to defy the notion of “those who cannot teach.” The multilingual culture in which the T&I profession is embedded has, in fact, altered T&I materials, techniques, and reception, as well as interruptions to T&I practice (Meylaerts, 2013). Professional knowledge and expertise are seen as vital for effective training. On the whole, T&I teachers are full-time university faculty with university jobs as their primary source of income (Torres-Simón and Pym, 2016).

Translation and interpreting teachers, in other words, are expected to take on a range of professional roles and devote substantial time to each. They must prioritize their roles and manage their time to meet the expectations of their students, institutions, and jobs. These roles, as well as the significance placed on each role, influence their professional development (Garner et al., 2016). A practitioner role may improve researcher performance if the teacher can develop a research topic from on-the-job experiences and subject it to methodical exploration (Shlesinger, 2009), whereas a trainer/educator role may impede their research activity if performing training tasks divert time and energy away from conducting research (Yang et al., 2021).

It is especially important to examine T&I teachers’ role identity in integration as it is linked to their professional development. Orlando (2016) frames the problem of role identity conflict as he stood “at the crossroads of practice, research, and teaching,” but through years of experience and reflection, he realized the need of merging the disparate roles as they inform one another in a cyclical way (p. 11). In particular, the integration of three roles will contribute to the flexibility needed to prosper in T&I teacher development, allowing for dynamic interaction across roles (Garner et al., 2018). In contrast, T&I teachers will be less inclined to invest in professional development activities if they are not identified as T&I teachers or do not feel acknowledged as trainers/educators, researchers, or practitioners.

There is a dearth of interest in addressing the issue of T&I teacher identity, compared to the strong tradition of published research on language teacher identity (Massey, 2019, p. 385). In fact, since T&I are essentially rendering what is said or written into another language, T&I teachers presuppose language teachers, whose identities have been well established. A common thread of the fruitful discussion on language teacher identity is the issue of NNS teachers forming and negotiating their NNS identities in educational contexts, which entails identity conflicts that NNS teachers experience as they work to balance many roles with varying meanings, norms, and expectations (Kayi-Aydar, 2019; Sung, 2022).

However, what distinguishes T&I teachers from other language teachers are different teaching competencies, goals, and profiles. Other than language competence, teaching skills, and disciplinary knowledge that one expects of a language teacher, T&I teachers are expected also to acquire first-hand personal experience of T&I profession and knowledge of market trends (Gouadec, 2010, p. 365). The ultimate goal of language teachers is for students to acquire some linguistic skills, but training future professionals for T&I teachers is the priority (Orlando, 2016, p. 52). T&I teacher profiles differ from other language teacher profiles in that they must include professional T&I practice, the academic discipline of translation studies, and teaching skills (Kelly, 2008, pp. 105–106). Hence, frameworks for comprehending language teacher identity cannot be simply applied to the T&I teacher population.

This study presents a T&I teacher role identity (TITRI hereafter) framework to illustrate how the main role of a T&I teacher influences three sub-roles and how these sub-roles interact. The TITRI framework is based on prior models of identity formation, particularly the Dynamic Systems Model of Role Identity (DSMRI) (Kaplan and Garner, 2017), and is used to clarify the interactions between and among sub-roles as well as between the main role and sub-roles. To be specific, the DSMRI serves as a framework for conceptualizing role identity and the dynamic interplay across roles. The proposal of the TITRI framework will add to the currently scant literature on T&I teacher identity research, shedding light on how different role identities may interact throughout the professional careers of T&I teachers. By extension, the framework may also foster an awareness of the impacts of higher education on T&I teachers and, as a result, provide grounds to offer suggestions for universities in China to encourage T&I teacher professional development.

## Theoretical background

### Translation and interpreting teacher professional role identity: Main role and sub-roles

Roles may vary from formal (a doctor, manager, or lawyer) to informal (a parent, spouse, or friend). People in today’s society take on a variety of professional roles, including teachers, doctors, and lawyers, all of which are context-dependent and may change over time. The expression of a teacher’s professional identity is how they carry out their professional roles, and this becomes the core of their teacher identity (Beijaard et al., 2004; Farrell, 2011).

Professional role as a concept is defined by Lunenberg et al. (2014) as “a personal interpretation of a position based on expectations from the environment and a systematically organized and transferable knowledge base.” The word “transferable” implies teachers’ ability to make theoretical knowledge explicit (p. 6). Professional roles are therefore defined both explicitly and vaguely, with different labels used in various settings. Each professional role can encompass different sub-roles (Garner and Kaplan, 2019). Terms such as “main identity” and “sub-identity” are used often in the literature to indicate the multiple, multi-faceted, and multi-dimensional nature of teacher identities that are constructed in various figured worlds at various moments (refer to Swennen et al., 2010; Akkerman and Meijer, 2011; Popper-Giveon and Shayshon, 2017). For example, Lunenberg et al. (2014) review relative related literature and identify six roles with different critical features for teacher educators—teachers of teachers, researchers, coaches, curriculum developers, gatekeepers, and brokers. Farrell (2011) believes that ESL teachers can house sub-roles as entertainers, cross-cultural experts, and oral interviewers, and Pellegrino (2009) holds that a music teacher’s professional role consists of a teacher and a performer so that the identity formation concerns the connection between performer and teacher selves. Similarly, Swennen et al. (2010) identify four sub-roles for teacher educators, namely, first-order teachers, second-order teachers, teachers in higher education, and researchers. Instead of stopping at the conflicts and confronts between these professional roles, they depict a cross-section of these four sub-identities, emphasizing transformation between the roles.

**FIGURE 1:**
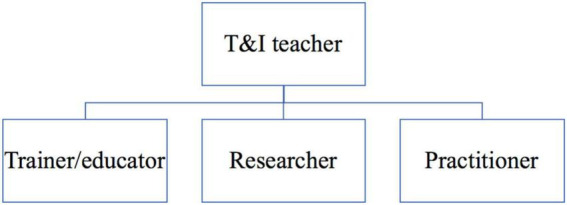
Main role and sub-roles of T&I teachers.

In the present study, the professional roles of the T&I teacher can be considered as a main role housing three sub-roles (Figure 1). The reason for this conceptualization is that the knowledge structure of T&I teachers in China consists of knowledge of teaching, research, and T&I profession (Li and Zhang, 2011; Kang and Shi, 2020). Accordingly, being a “well-trained, professional, and competent” (Wu et al., 2019a) T&I teacher requires one to be a T&I trainer/educator, researcher, and practitioner at the same time. On the contrary, trainer/educator, researcher, and practitioner roles serve as the elements to construct the main role of the T&I teacher role identity.

### Understanding translation and interpreting teacher professional role identity: An integrated framework

The meanings for an integrated framework are twofold. First, such a framework helps to clarify how different sub-roles interact and how they relate to the main role. Even within T&I studies, it was only until recently that attention was paid to empirical research on T&I teachers and their professional development (Li, 2018; Wu et al., 2019b). Kang and Shi (2020) proposed the Practice-Teaching-Research (PTR) model as a three-in-one approach to understanding T&I teachers’ positioning, whereas Wu et al. (2021) studied the identity and emotions of a novice T&I teacher. Despite identifying the three dimensions of T&I teacher obligations, neither study has investigated the dynamic and complex interaction between roles and how this interplay affects professional development.

Second, though tensions and conflicts exist between the main role identity and sub-role identities or between sub-role identities, it is believed that individuals seek increased coherence, alignment, and integration within and between roles in the professional development process within a relatively long time (Burke and Harrod, 2005). Individuals’ investments in certain roles are mediated by varied role identities they construct in specific contexts (Sung, 2019), which means that T&I teachers prefer to invest significantly in role identities that can be negotiated while refraining from those that cannot. For example, the practice-oriented nature of the T&I field expects T&I teachers to be active practitioners to promote training/educating (Kelly, 2008), but the nature of higher education expects T&I teachers to invest more in research production. These two contrasting expectations may produce identity conflicts for T&I teachers. In the long run, however, if the roles are put in a coherent framework, it would be direct to understand how these role interactions influence the whole professional identity development.

### Dynamic systems model of role identity

**FIGURE 2:**
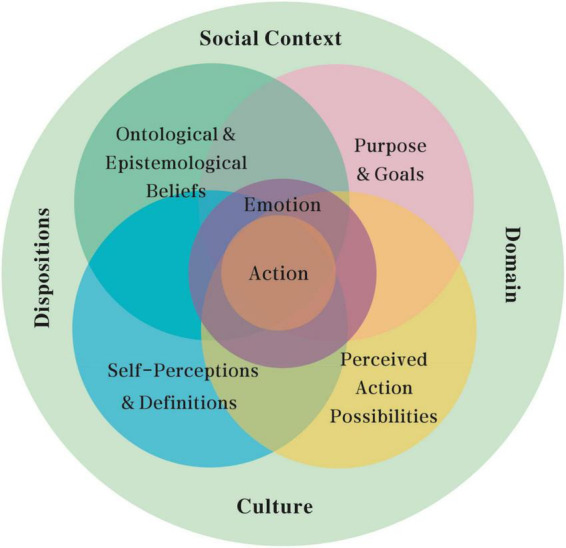
The dynamic systems model of role identity (DSMRI) (Kaplan and Garner, 2017, 2018; Garner and Kaplan, 2019).

As an “emerging model” of teacher identity (Beijaard, 2019), the DSMRI (Kaplan and Garner, 2017, 2018; Garner and Kaplan, 2019) takes role identity as a unit of analysis and conceptualizes role identity as an emergent, complex, contextualized, and dynamic system anchoring in “action,” a system that encompasses four interacting components and locates them within a control parameter consisting of socio-cultural context, subject domain, and dispositions (Kaplan and Garner, 2018). Adopting a complex dynamic system (CDS) paradigm connoting that the continuous, interactive interaction of interdependent elements contributes to the whole system’s behavior (Waldrop, 1993), the DSMRI is then set to bridge the dichotomies of identity research by integrating both personal and collective, stable and changing, and conscious and unconscious aspects of identity development (Vignoles et al., 2011) into a coherent, theoretical framework (refer to Figure 2).

The core structure of the DSMRI consists of four contextual-constructed, reciprocal, and partially overlapping components: (1) ontological and epistemological beliefs relate to the knowledge and conceptions that are held to be true by teachers about the world and the subject domain and beliefs about the certainty, complexity, and the credibility of their ontological knowledge, along with the emotions connected to these beliefs (Kaplan and Garner, 2018). For instance, a T&I teacher may believe firmly that he/she will be a successful trainer/educator only if he/she has enough translation practice experience but may view with doubt that T&I research experience has a bearing on T&I training/educating; (2) self-perceptions and definitions concern both T&I teachers’ knowledge and emotions regarding his/her personal and social attributes and characteristics relating to T&I teaching, research, and practice. A T&I teacher may define himself/herself as curious, talented, or studious, may perceive himself/herself to be keen in teaching or practicing but unenthusiastic in researching, and may identify himself/herself into a particular social group, such as “intellectuals”; (3) purpose and goals refer to T&I teachers’ overall purpose and more concrete goals for training/educating, research, and practice. These goals are both long term and short term, the dimensions of which can vary from intrinsic to extrinsic, personal to collective, and general to specific (Butler, 2007); and (4) perceived action possibilities include strategies and behaviors that T&I teachers feel available for them to perform in the roles of teachers, researchers, and practitioners but exclude those actions that they perceive as inappropriate and impossible for them (Kaplan and Garner, 2017).

On the whole, these four components interact dynamically to form the basis for decision-making and actions in the role, and *vice versa* (Kaplan and Garner, 2018; Garner and Kaplan, 2019). For example, a novice T&I teacher had a particular set of pre-existing beliefs about the training/educating such as it was boring to give students “standard translation answers,” which might align with her professional goals of empowering students to develop learning autonomy. The teacher held that a self-definition as both a transmissionist and a transformationist led to her action possibilities of presenting translation training as merely displaying facts or as a negotiating process (Wu et al., 2021). In turn, taking actions in a certain role impacted the four components of the DSMRI system. For instance, the change from teaching GE (general English) to teaching ESP (English for Special Purpose) impacted the teacher’s beliefs about ESP as more need-driven and genre-specific than GE and self-perceptions about confidence in teaching expertise and competence, thus bringing changes to more domain-specific English-learning goals, leading to further action possibilities of learning and teaching (Jiang and Zhang, 2021).

The structure portion of the DSMRI is one distinctive aspect that is relevant to this research to conceptualize the interplay of different roles. The structure part of the DSMRI is used to represent the harmony or tension between different elements within each component, the alignment or misalignment between different components of the same system, and the integration or disintegration of different DSMRI systems, either for different roles or for different individuals (Kaplan and Garner, 2017). Positive emotions are more likely to develop when there is harmony and alignment, while negative emotions are more likely to appear when there is tension, misalignment, and disintegration (Wang et al., 2021).

The DSMRI is not entirely new. After Kaplan and Garner put forward the framework, several studies disseminated the DSMRI by exploring the potential of being a theoretical framework to understand professional identity development. For example, Hathcock et al. (2020) utilized the DSMRI to analyze the dynamic interplay between science teacher role identity and other role identities within a professional development context and concluded that alignment or tensions within and between these role identities either promotes or impedes action possibilities. In a study of EFL teachers’ identity change, through investigating teacher learning and identity as a DSMRI, Jiang and Zhang (2021) classified three prototypes of identity change—significant change, moderate change, and minor change. They also proposed an assumptive model to understand teacher learning and identity change in the reform context. Duan et al. (2021) applied the DSMRI in a quantitate way, constructing a four-factor (i.e., social, role, target, and action perceptions) index of social work, professional identity, and identifying differences between different genders, backgrounds, and certifications, etc.

## The study

Drawing on the DSMRI, T&I teacher role identity can be conceptualized as four interrelated components of beliefs, goals, self-perceptions, and perceived action possibilities. The DSMRI allows extraction and explanation of sub-roles, and their interplay as the T&I teacher is the main role that harbors the sub-roles of trainer/educator, researcher, and practitioner. This model thus enables a holistic and thick description of how different role identities develop, interplay, and result in changes in role identity (Garner et al., 2018).

The present study is a multiple-case narrative study, a qualitative research strategy that draws on narrative data collected with multiple people as part of a research project (Shkedi, 2005, p. 20). By treating each participant as a case, this study can analyze data within and across settings (Baxter and Jack, 2008). The investigation starts through a narrative approach, a way of “thinking about, and studying experience” following a recursive and reflexive process (Clandinin and Huber, 2010). Narrative in this study is not only a means of understanding human experience and meaning but also a means of human expression (Shkedi, 2005, pp. 11–12). To be specific, narratives frame our understanding of T&I teacher role identity as it provides an interpretative tool to construe teachers’ professional purpose and goals, beliefs about T&I training/educating, research, and practice, and perceptions of related roles and how this is connected to the overall professional identity of a T&I teacher.

The advantage of multiple-case narratives over a collective case study is that it has the potential to examine a greater number of case narratives while the edge over case survey lies in that it consumes primary “raw” data instead of secondary-interpretive data and thus preserves the narrative-qualitative nature (Shkedi, 2005, p. 26). The choice of multiple-case narrative approach/inquiry enables the study to encompass as many case narratives as needed, while making possible a more in-depth analysis of each T&I teacher role identity. Moreover, multiple-case narrative affords a ground for generalization (Shkedi, 2005, p. 25), a less discussed topic by qualitative researchers (Polit and Beck, 2010). The analytic generalization that this study aspires for means that we generalize from specifics to a broader concept or theory (Firestone, 1993; Shkedi, 2005, p. 192; Yin, 2018, p. 99). In practice, we made use of the DSRMI as a theoretical-conceptual framework to guide the data collection and analysis. Then, the findings were translated into theoretical analysis so that the case narratives serve to illuminate larger issues and, thus, issues of general significance (Marshall and Rossman, 2014).

Our methodological goal is an analytic generalization that is a conceptual level higher than the collection of multiple-case narratives, leading to deeper insights into T&I teacher professional development in China. Specifically, this study set out to address two research questions:

(1) How do the three sub-roles of a T&I teacher (T&I trainer/educator, researcher, and practitioner) interact with each other?
(2) How do the three sub-roles relate to the main role of a T&I teacher?

### Research setting and participants

In a multiple-case narrative study, the primary way to investigate any phenomenon is through the experience of the individual (Shkedi, 2005, p. 41). Yin (2018, p. 124) emphasizes the necessity of carefully selecting individual examples so that similar or contrasting results can be expected, noting that purposeful rather than random sampling should be used. The current study’s target population is T&I teachers in China. T&I teachers are defined as teachers who are in-service teachers in Bachelor of Translation and Interpreting (BTI) or Master of Translation and Interpreting (MTI) institutions sanctioned by the Ministry of Education in China (Ministry of Education of the People’s Republic of China, 2017). Thus, participants in this study were purposefully chosen because they: (1) had professional histories under investigation, which means they were in-service T&I teachers with experiences as trainers/educators, researchers, and practitioners; (2) felt comfortable being interviewed and observed and could articulate their experiences; and (3) agreed to spend the time necessary with the researchers and were not frightened by too much self-exposure (Shkedi, 2005, p. 42).

In deciding the case number, Yin (2018, p. 124) states that the numbers 6–8 is most likely to provide “compelling support” for both literal and theoretical replications. The study, therefore, restricts the participant number to 7 since less than 6 limits the understanding of T&I teachers as a professional group and more than 8 risks sacrificing a detailed and in-depth analysis.

Another sampling strategy adopted is the maximum variation in terms of T&I teachers’ individual characteristics to gain commonality across diversity and enhance transferability (Merriam and Tisdell, 2015, pp. 257–258; Patton, 2015, pp. 428–429). The present study maximized the participant pool by including both male and female participants aged between 20s to 50s, with different years of teaching experiences, of different educational backgrounds, and working in different types of universities. In mainland China, universities are roughly classified into Tiers 1 and 2. Furthermore, translators and interpreters normally have their preferred working language pair, with one being their mother tongue. Table 1 presents profiles of the seven research participants, whose names are pseudonyms.

**TABLE 1:** Research participants.

Name	Gender	Years of being a T&I teacher	Professional title	Highest educational qualification	Types of university	Working language pair
Amy	Woman	7	Lecturer	MTI	Tier 2	Chinese–English
Gary Betty	Man Woman	11 14	Associate Professor Lecturer	PhD in economy MA	Tier 2 Tier 1	Chinese–Japanese Chinese–English
Carol	Woman	15	Lecturer	Ph.D. in Translation Studies	Tier 1	Chinese–English
Dina	Woman	15	Associate Professor	Ph.D. in English Language and Literature	Tier 1	Chinese–English
Keven	Man	22	Professor	Ph.D. in Translation Studies	Tier 1	Chinese–English
Paul	Man	27	Professor	Ph.D. in Linguistics	Tier 1	Chinese–English

### Data collection and analysis

In-depth interviews were the primary source of data as the study investigates T&I teachers’ own stories in their own words. Since there are no hard and fast guidelines regarding the optimal number of interviews in multiple-case narratives, this study followed the standard practice of conducting two rounds of interviews (Shkedi, 2005, p. 62). The first round of interviews started with participants’ background information, such as biographical information, and educational qualifications and moved on to their decisions to become T&I teachers. Participants were asked to discuss their experiences and difficulties as a trainer/educator, a researcher, or a practitioner. Interview questions centered around self-perceptions, goals, beliefs, and action possibilities of the DSMRI. The second round of interviews took place approximately 1 week later (or the participants’ convenient time) to ensure the narratives fully cover each role identity of the T&I teacher and to clarify any follow-up doubts or questions. The interviews were audiotaped and each lasted between 60 and 120 min. Less than 60 min for an in-depth face-to-face interview is too short for participants to reflect on their experiences and engage in the meaning-making process (Flick, 2018, p. 25), and more than 120 min is likely to exhaust both the researcher and the participants (Shkedi, 2005, p. 62). Due to the COVID-19 lockdown, the interviews were initially scheduled to be done face to face but were subsequently modified to audio or video calls. Interviews were carried out in the participants’ native language, Mandarin, with selected excerpts used in this article translated into English by the authors.

The ultimate goal of data analysis in this study was to “re-tell” the multiple-case narratives using the DSMRI language and structure. The whole study was carried out cyclically and iteratively (Huang, 2013, p. 116), with the researcher moving between the obtained data and the theoretical framework (Trent, 2013). In practice, this included using the role as the unit of analysis and the DSMRI components to code the narratives, synthesize and integrate the coding, and discover themes based on the DSMRI assumptions about the content, structure, and process. This iterative process was repeated until research questions could be addressed. In establishing trustworthiness (Creswell, 2013, p. 250), maximum variation was undertaken to include more diversified characteristics and different backgrounds of the participants to ensure transferability. Moreover, the researchers aimed to describe in a rich, thick, and detailed way (Shkedi, 2005, p. 190) so that the audience can have enough information to determine the match between the case described and their own. For dependability, member checks took place between the two authors and between the two authors and the research participants.

### Researcher role

In qualitative research, it is essential that researchers position themselves to be both “a part of and apart from the world observed” (Patton, 2015, p. 612), which also reveals the concept of “reflexivity” (Creswell, 2013, p. 223). The first author has been a T&I teacher in a mainland Chinese university for more than 10 years. This personal experience as a T&I teacher has rendered the study an insider perspective (Huang, 2007, p. 84, Huang, 2013, p. 19; Merriam and Tisdell, 2015, pp. 146–147), which has influenced and informed the design of the present study. However, the researcher is also fully aware that as an insider, her involvement, immersion, and empathy (Shkedi, 2005, p. 47) reflect her academic philosophy.

The researcher’s relationship with the participants shapes the narrative stories in some ways. Reciprocity between the researcher and the participants, which requires intense sharing, trust, and mutuality, is vital for high-quality qualitative research (Lincoln, 1995, as cited in Creswell, 2013, p. 259). The first author thus maintained regular contact with the participants to know about their updates through social media. Besides, this study deliberately chose half of the participants (3–4) who had a relatively long-term personal connection with the researcher, be them the researcher’s colleagues or school fellows, and the remaining half only had professional acquaintances whom the researcher had met in some nation-wide T&I teacher education conferences or academic workshops. Hence, the researcher’s background as a T&I teacher in mainland China, together with their personal connection with half but not all participants, afforded a balance between subjectivity and objectivity (Duff, 2008; Trent, 2013).

## Results

### Between trainer/educator and researcher roles: “They help each other”

Teaching and researching are considered two interrelated components that both bring challenges for T&I teachers (Orlando, 2016). When talking about the relationship between the roles of trainer/educator and researcher, most participants recognize both as the major obligations for T&I teachers and can gain insights and skills from both in a meaningful and reciprocal way.

The majority of the benefits they identified from research can be associated with tangible pedagogical effects, like what to teach, how to teach, and how to test in a T&I course. Gary, for example, explained how he used research to help students to solve translation difficulties.

#### Extract 1

*The differences between the two languages (Chinese and Japanese) sometimes mean there are some untranslatable terms. When you are teaching, you need to explain to your students. It is at this time that I need to look up some research articles to guide me to solve problems.* (Gary)

These tangible pedagogical effects also included using research as a tool to “better define training difficulties” and as “a yardstick to judge the training effects.” Besides tangible pedagogical effects, a central meaning of research seems to fall into the category of “reflection” (Postholm, 2008), thinking in new ways of perceiving things from alternative perspectives.

#### Extract 2

*For one thing, research is a guide in your training mentality and methods. What we teach and how we teach should not be purely experiential. It is not just only what you think is good that can be 100% passed on to your students. This is not scientific enough. For introducing a new training mentality and methods, you need to be equipped with research achievements. Furthermore, research can provide a means to better understand your training*. (Keven)

It is worth noting that when reflection does not merely stop at a thinking level, it can be a means to connect theory and practice, i.e., a means to integrate the two sub-roles of T&I trainers/educators and researchers. It leads to inspiration for new research ideas, new projects, or new courses, thus empowering their T&I trainer/educator and researcher identity.

#### Extract 3

*One course I taught was called “Conference Interpreting as a Profession,” which included the concept of the market. If you are going to introduce the T&I market, you need to start with the language service sector. The T&I market is just a small yet important part of the sector. This intrigued me to initiate a research topic about the language service sector. But for the course, I would not have initiated such a project. I am very interested in it now.* (Carol)

Instead of merely stopping at what training has contributed to research and *vice versa*, Paul and Amy went one step further to create a virtuous cycle between the two roles. Paul not only utilized research to address problems in class, but he went on to compile his textbook by incorporating research results and his knowledge of the T&I curriculum. He expanded his research by using his textbook as instructional materials and bringing examples from his study into classes to make theory less abstract and practice more alive for students. In doing so, he created a virtuous cycle between T&I training and research. Similarly, Amy, a doctoral student, though still fighting for her threshold of being a researcher, believed that these two roles “help each other.”

#### Extract 4

*I really believe these two roles help each other. Before doctoral study, I might have simplified all the problems arising from T&I course. I just copied others’ successful training experience without modifying much [*…*] Now that I understand there should be a more scientific way of referring others’ classroom practice and more systemic way of organizing my training plan. On the other hand, in my training, I noticed that students’ autonomy in learning is at a relatively low level. For students in my school (a local university), they simply don’t want to study and don’t know what to do and how to do it. I want to pay more attention to this and make a research topic out of this. I think it will be very promising in research area*. (Amy)

Amy referred to research as a syllabus design repertoire for her T&I training. When she initiated the research plan, she tended to bear her students’ learning in mind. Her desire to incorporate both research and training offers the possibility that she will be open to engaging in the process of doctoral training, which is a necessary step for enacting the role of a T&I researcher.

### Between researcher and practitioner roles: “A yawning gap”

When asked about the relationship between research and practice, most T&I teachers reported no direct help from research to their practice and *vice versa*.

#### Extract 5

*I don’t perceive much relationship between research and practice, at least not strongly related. When we are interpreting, it is almost impossible to think about how to use some theories or think of some research results at that very moment. It might not be 100% true to say so as you may already be equipped with the T&I theory deep enough to change your mindset. What I mean is personally speaking, I tend not to consider those theories when I am interpreting*. (Keven)

#### Extract 6

*[*…*] Well, this kind of practice convenience (brought about by linguistic perspectives) does not necessarily lead to rich research output. My broad interests and open mind in translation practice without pre-constructed categories often distract me from one research topic to another, consuming too much time and energy and sometimes resulting in zero paper.* (Paul)

The belief that T&I practice is not much relevant to T&I research confirms what Chesterman and Wagner (2004, p. 1) term as “a yawning gap” in the T&I discipline. They went on to highlight that, when compared to other fields, few professions have such a significant divide between theory and practice and between researchers and practitioners. We chose and compared interview data from the same individual to better explain the disparity. As a result, these descriptions give a thorough and clear comparison of the two roles (Table 2).

**TABLE 2:** Misalignment between T&I practitioner and researcher roles.

DSMRI Component	T&I Practitioner	T&I Researcher
Self-perception	It is super-high. Like an old hand, you really miss the feeling of interpreting if you leave the profession for a long time. (Dina)	The feeling of rejection is much crueler than you were turned down by some handsome boy when you were young. The most difficult part is that after you got rejected, how could you possibly carry on? (Dina)
Belief	I fulfill the interpreting task without theory anyway. (Amy)	I know that T&I research in China starts late and absorb inspiration from linguistics. For me, I am not sure whether the T&I theory is important or not, and also not sure whether I need to clear out the theories and put it into practice. (Amy)
Goal	The only thing you need to consider is how to interpret and how to interpret better. It is instant. (Keven)	For producing research, you have to find some cases, which you cannot solve in your interpreting practice, and you analyze, and write the thesis. It is a long process. (Keven)

We can observe from the table that distinct, or even opposing, DSMRI components are associated with the T&I practitioner and researcher roles. Practitioners are described as being emotional “super-high,” but researchers are described as being “cruel.” These opposing phrases for emotions imply that the T&I practitioner experience is joyful and relaxed, whereas the researcher is more irritated and painstaking. Furthermore, the nature of labor as experienced by our participants varied greatly. The work of T&I practitioners was believed to be “fulfilling the task” and “instant,” while research work was to “find cases, analyze, and write the thesis” and thus “a long process.”

This yawning gap between T&I practitioner and researcher roles, which causes unwillingness or reluctance for integration action possibilities, is recognized by participants. Dina said that a lack of time may be a big barrier to future integration of action possibilities.

#### Extract 7

*I have a wish to collect all the interpreting practice that I can to make it into a corpus, a big one. I really try my best to audio- or video-tape all the conferences. What happens in the end? You have collected more and more corpus, fully loaded in every stockpile. Yet you don’t have the time to sort it out and organize it into one or more research ideas. You simply don’t have time. To do that, you need a period breaking away from other tasks. [*…*] It (practice experience) just offers an opportunity to collect data, but whether it can be turned into meaningful research actually depends on the time and energy invested*. (Dina)

Though it is the appeal of existing literature that, ideally, T&I practitioners and researchers should endeavor to develop deeper ties, the opposing and contrasting DSMRI components foreshadow skepticism and unwillingness to cross the divide. Furthermore, there are frequent complaints about a lack of time for T&I practice in the comments, implying that the other two roles take precedence over T&I practice. The secondary place of T&I practice is evident in the following excerpt.

#### Extract 8

*I fully stop my T&I practice at the moment. I cannot do so many things at the same time. I need long periods of time to do my research so that I have to give up practice for now. [*…*] The materials I use for class is from my past experience, like two or even three years ago. You know the T&I market is changing rapidly. I am very dissatisfied with my training materials but I have no other alternative. I need to wait until I get my PhD degree to devote into T&I practice*. (Betty)

### Between trainer/educator and practitioner roles: “A role model for students”

An important theme developed from the relationship between T&I practitioner and trainer/educator roles is that the former helps make sense of the latter in both a direct and indirect way. T&I teachers were committed to using their practice as an important source of training. They were concerned with offering students a good training source, ranging from pre-translation preparation to up-to-date first-handed materials to after-translation reflection and to the cultivation of students’ professional skills and knowledge. They also emphasized the need to integrate simulated T&I tasks into the course.

#### Extract 9

*We (T&I teachers) have a well-established tradition of audio recording the conference interpreting scene and use in our training course, which of course can only be done with employers’ permission. Actually, they (the employers) welcome and support this as they know we are training tomorrow’s interpreters. From our perspective, the fact that we as practitioners fulfill the task means we have already processed the whole interpreting task, from pre-task preparation, pre-site preparation, on-site experience, problem-solving during the task, and customer feedback to after-task reflection. This experience, when transmitted to the training process is well-received by our students*. (Carol)

As T&I practitioners and trainers, they stressed that their training must be scientifically updated so as to meet the “live” needs of T&I tasks besides focusing on professional competence and skills, avoiding merely giving “didactic recipes.”

#### Extract 10

*This (using on-site T&I practice materials for training) is definitely one major feature of T&I training. Besides national conference materials, those T&I materials for local economic entities proved to be especially useful. Just think about most of your students will serve local economic entities in the future. They thus perceive these on-site experiences as highly relevant [*…*] For instance, you can ‘transport’ what you have encountered as difficulties into a classroom and ask about their response. This will be an interesting discussion about interpreting decision-making process*. (Dina)

Translation and interpreting trainers/educators paid special attention to the way that they behaved as T&I practitioners, revealing the awareness of the special profession they were training/educating. The alignment among their training goals of integrating on-site T&I experience, epistemological faith in ontological beliefs in authentic training materials, and their action possibilities of engaging in both training and practice reflect a high level of integration of their role identities as trainers/educators and practitioners.

Other than perceiving a direct link between T&I training and practice, they also referred to an indirect link concerning the professionalism of future T&I practitioners.

#### Extract 11

*Students like to hear your personal stories as interpreters. What’s more, if they become interpreters one day and happen to meet some employers who know your name, they will be greatly inspired. They feel that they come from an outstanding group, in which they themselves are part of it. I think our own practice of excellence will be great incentive for our students*. (Dina)

From the excerpt, Dina was dedicated to the T&I field. She discussed her professional role identity as an interpreter and pride in the profession, both of which are essential constructs of T&I professionalism (Liu, 2021). Other than transferring T&I experience directly to inform T&I training, they also passed on their knowledge about the profession and attitude toward professionalism. Adherence to ethical practice was another dimension of T&I professionalism that they wanted to impact their students through the training course. Carol, in the following except, believed that future interpreters should be sensitized and trained to deal with the boundaries and criteria of interpreting in the real world.

#### Extract 12

*I want my MTI students to know that they are not undergraduates anymore, instead, they are future practitioners in the field. [*…*] It would be a waste if they are not to be a practitioner in the field. So I tell them ‘if you don’t comprehend, please do not make it up,’ or else it will be a serious ethical issue. You will lose your job for violating the code of conduct*. (Carol)

Carol not only instructed her students about professional codes of ethics but also provoked students with a profound understanding of ethical issues in interpreting. As an interpreter working for experts during COVID-19, she further stated that she held herself to be “a role model” using her practice experience during the pandemic, hoping to provide a positive impact by making future interpreters reflect upon their “accountability” to the community and the humanity at large (Baker and Maier, 2011).

#### Extract 13

*Now looking back (at being an interpreter during COVID-19 period), I want my students to know that there need to be someone to do something at that very moment. If you, as their teacher, cannot perform the task, how can you expect your students to do so? [*…*] If my work can at least inspire some students who want to be devoted to T&I practice, I will consider it as rewarding or meaningful.* (Carol)

From the excerpt, Carol is held responsible for the consequences of her interpreting practice and reflected carefully about her decisions and the impact. Instead of simply prescribing the codes, she would like to incorporate them into the training curriculum to provide her students, trainee interpreters, with a platform to reflect on various issues and situations that they may face in their professional lives in the future, allowing them to examine their own professional values, becoming more conscious of and critically assessing the values. The merging of the T&I practitioner and trainer roles opens up the action possibilities of transforming the training classroom into what Baker and Maier (2011) refer to as “a protected environment of experimentation and reflection.”

However, the interaction between trainer/educator and practitioner is not only mutually beneficial but it also indicates a contradiction in higher education, where a T&I teacher is expected to fulfill the institution’s mission of educating students rather than performing T&I practice. T&I teachers often receive the message of prioritizing their researcher and trainer/educator roles over their practitioner role. For example, while being fully aware of the necessity of T&I practice, Paul persisted on making it the last priority due to limited time and energy.

#### Extract 14

*I will put training/educating in the first place. This is what we are required to do and get our monthly salary for, right? After we fulfill the teaching task, we can arrange our other two missions. [*…*] T&I practice is vast and never-ending, sometimes interrupting your research process. Though I believe the time for practice is important, but it really takes time. (Paul)*

Likewise, Carol indicated a similar difficulty and ranked the role of the practitioner as the last consideration.

#### Extract 15

*I have to deal with three roles, which are all highly demanding. I have no other alternatives but to set priority under limited time and resource. My first priority is training/educating, which needs to be fulfilled before other considerations. And I devote the rest into my research to try to fulfill research demands. Only with these two accomplished, I would be able to consider practitioner role*. (Carol)

Therefore, it is evident that, that even though T&I teachers displayed great interests in practice, they prioritize their trainer/educator and researcher roles over their practitioner role.

### Between the main-role and sub-roles: “1 + 1 + 1 < 3”

Within the context of the higher education system, T&I teachers are identified strongly as college teachers. Compared to other job opportunities, T&I teachers perceived a teaching post in tertiary education as what they are meant to do, referring to the fact that it was their “ideal job.” This is also demonstrated that, when asked about their professional future, none of the participants would consider leaving the teaching profession.

#### Extract 16

*When I was a college student, I envied my teacher for their flexible working hours. They always seemed to enjoy their free time without following a traditional nine-to-five working routine. Since that time, I have had a firm belief that a college teacher is an ideal job for the future*. (Amy)

#### Extract 17

*I have had a very firm belief to be a teacher since I was still a student of English about 30 years ago. All through these years, my determination to be a good teacher has always stood steady. I devoted all my life to it, without even considering another career path*. (Paul)

Nevertheless, T&I teachers experienced tensions among the three roles and responded to them in different ways. When asked about the most difficult part of bringing together the three sub-roles, most participants noted a lack of time and energy due to a heavy workload. The workload ranged from a lot of teaching-related tasks, such as preparation brought by online courses during the COVID-19 period, teaching extra classes, staff meetings, and interviews for MTI potential students, to research-related obligations. Betty, for example, mentioned that it was a complete failure to juggle research, teaching, and practice, calling it a “vicious circle.”

#### Extract 18

*Doing research takes up a long period of undivided time and attention. For example, if I can read for 5 or 10 days in a row, I will be very efficient and productive. But it is just an ideal situation. Last week, for example, I was requested to act as an interviewer for prospective MTI students, which takes 2 days. After that, you must rest for a whole day to recover from two exhausting days. Three days have gone with little progress in research. Because of this kind of workload, you can barely afford the time to take up any T&I practice, which will cause a predicament in training as you don’t have the up-to-date materials for your students*. (Betty)

Dina’s feeling of contradiction was triggered by her attempt to integrate the three roles. Instead of coherence, she felt a disintegration among the roles. This was caused by her research plan which was not in congruence with her training and practice. Instead of mutually beneficial to facilitate professional development, she believed “1 + 1 + 1 < 3.”

#### Extract 19

*It (T&I practice) is super high. If you quit the profession for a long period, you will miss the thrill of translating like an experienced hand. As a result, there is a conflict between competing priorities. On the one hand, you work in a university and must dedicate your time to teaching. On the other hand, if you want to do effective research, you must commit your 100% attention. You should wake up at 6 a.m. and work your way through sorting, analyzing data, writing, and maybe submitting. Only 30 or 40% of attention cannot make a good research, at least for me. And if you put your whole heart into research, you won’t have time or energy to devote to interpreting. For me trying to do three at the same time makes it worse, after all, 1* + *1* + *1* < *3*. (Dina)

Translation and interpreting teachers repeatedly mentioned that they are aware of the significance of research to their career advancement and the pressure is more likely to come from an institutional level. This institutional level of pressure together with the fear to lose their jobs, which in other words, imposed a sense of insecurity for not accomplishing research tasks.

#### Extract 20

*I work in a comprehensive university. At the very beginning, there were no special research requirements for a T&I teacher, a practice-oriented position. But this began to change in 2015. Even an interpreting teacher can not get a higher academic title by not doing research. What’s worse, if you cannot secure a higher academic ranking, you will be forced to leave the position. It is at this time I began seriously thinking and I realized how I love T&I teaching, and how I love to be a T&I trainer/educator. In order to keep my job, I have to do research, and most urgently, I need a doctoral degree. The road to becoming a Ph.D. student is full of twists and turns. Comparatively speaking, I feel much more at ease with my teaching and practice*. (Betty)

It is worth noting that a number of participants responded to a similar sense of insecurity about their tenure-track contract. Traditionally speaking, the “up or out” rule constitutes an important part of the academic tenure system in US universities, meaning that a permanent position is provided by the institution only to those faculty members who have earned an established record in teaching, research, and service. In mainland China, the “up or out” rule was introduced first in 1994 in Tsinghua University but was implemented in most Tier 1 and Tier 2 universities with varying criteria from 2000 (Shu et al., 2020). Participants from Tier 1 and Tier 2 universities are more likely to reveal their uneasiness at not being given a tenure contract, which helps to accelerate the disintegration among the roles.

In conclusion, T&I practice and their job satisfaction toward the main role T&I teacher are believed to bring the three roles together while heavy workload, laboring research activity, and job insecurity due to the tenure system are to separate the three roles.

## Discussion

**FIGURE 3:**
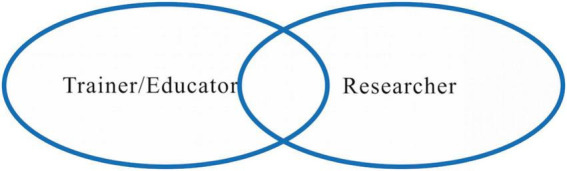
Trainer/educator and researcher role identity interaction as overlapping.

**FIGURE 4:**
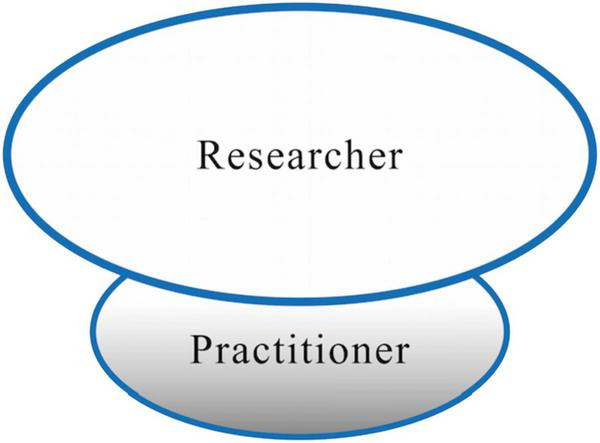
Researcher and practitioner role identity interaction as overshadowing.

**FIGURE 5:**
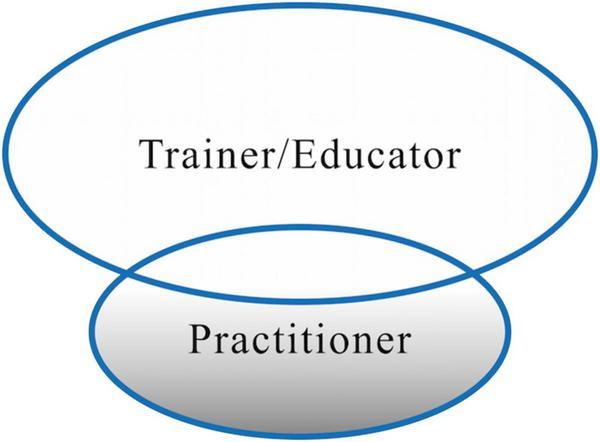
Trainer/educator and practitioner role identity interaction as overlapping and overshadowing.

**FIGURE 6:**
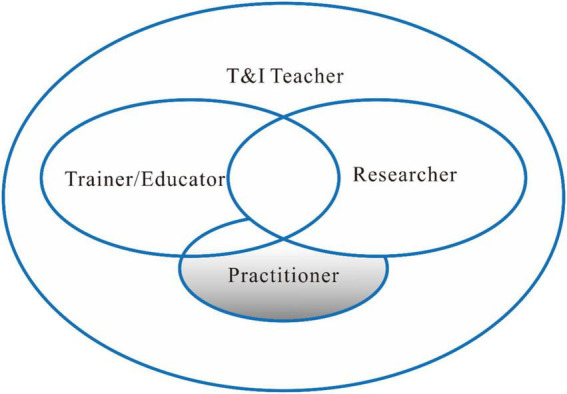
An overall TITRI framework.

In the light of the DSMRI (refer to Figure 2), which views role identity development as a dynamic and ongoing process, the discussion below begins with analyzing and visualizing the three kinds of dynamic interaction between roles (Figures 3–5), before arriving at an overall T&I teacher role identity (TITRI) framework (Figure 6). The unified TITRI framework conceptualizes the interaction and relation, extending our knowledge about T&I teacher role identity.

### Overlapping role identities: A combination of different roles

According to the findings, participants see both the sub-roles of T&I trainer/educator and researcher as significant duties for T&I teachers and can acquire insights and skills from both in a meaningful and reciprocal manner. Reflection is associated with the enlightenment or empowerment of their T&I trainer/educator and researcher role identities: it allows for the creation of new research ideas, initiatives, or training courses.

From a DSMRI perspective, their role as T&I trainers/educators exhibits existing beliefs and goals that form parts of their researcher role identities, and which are in harmony with their goal of fulfilling institutional requirements. For Carol and Paul, even before the research element was included in the training course, their existing self-perceptions and professional goals aligned with the goal of a T&I researcher role identity. The reflection process provides a platform to connect their beliefs and interests in teaching with the T&I researcher identity. The institutional requirements of teaching new courses were incorporated into the training/educating that helped carry out the research plan. What is more, the previous academic training they received as T&I researchers provides the skills and strategies for carrying out the research plan, facilitating them to enact the role of the T&I researcher.

Thus, the proposed TITRI framework uses two overlapping ovals to indicate two different yet mutually beneficial role relationships. In the framework, both trainer/educator and researcher roles are visualized as the same size, the interaction represents a large degree of overlapping (Figure 3).

The size and intersection of the circles vary based on different roles. The ovals may differ for each role, with some being larger or smaller depending on the prominence or importance of the role. The larger the oval is, the more prominent the role identity is. Between the ovals, there is more or less overlap, indicating the intersection between roles. The greater the overlap, the more integrated the role identities are.

#### Overshadowing role identities: Practitioner as secondary

Our findings indicate that the majority of teachers perceived the interface between the T&I researcher role and the practitioner role as a “divide” (Shlesinger, 2009; Orlando, 2016, p. 40). Despite their awareness of the need to reconcile each side and bridge the gap, the DSMRI components they displayed toward the two roles were fundamentally opposed, making the divide a challenge for the T&I teachers to overcome.

The interaction between the researcher and practitioner role is not only a divide (not overlapping), but also the former overshadows the latter, outlining a secondary position of T&I practitioner role (refer to Figure 4). Although T&I teachers expressed considerable delight and passion in their practitioner roles, the reality that promotion and incentive scheme primarily assess their research output leads them to transfer their primary focus to research while fighting to stay engaged in the profession.

The secondary position and overshadowing identities can be explained by the effects of the institutional context. From a role identity perspective, both researcher and practitioner roles are commonly enacted by T&I teachers after they enter universities. While a practitioner role may align with the teachers’ interests, bringing positive emotions such as excitement and enjoyment, they are also strictly defined by the institution that the T&I teacher serves. Higher education in China is undergoing an expansion and internalization process during the last two decades. First, China began its fast expansion of higher education in 1999, signaling a shift from an elite-oriented to a public-oriented higher education system, with increasing skepticism following suit about the quality of undergraduate education (Liu, 2013). As a response, the Chinese government carried out a series of quality guarantee measures to support the shift from quantity expansion to quality enhancement (Yin and Ke, 2017) for the purpose of establishing a modern higher education system with distinct Chinese characteristics (Han et al., 2020). Second, during the last three decades, China has articulated its ambitions of building world-class universities and developing first-class disciplines beginning from Project 211 (1995) and Project 985 (1998) to the “Double First-class” Initiative (2015).

Because of the expansion and internationalization of higher education in China, academic performance has become a primary goal for universities, posing strong washback effects on practice-oriented discipline teachers such as T&I teachers. To evaluate research performance and to strengthen internationalization, teachers are mandatorily required to publish in (S)SCI journals to obtain tenure and promotion. This is not unique in China though, as the tenure was proposed as early as 1940s and has been prevalent in North America (McPherson and Schapiro, 1999). However, what is distinct for China is that, for both Tier-1 and Tier-2 universities, with or without a tenure system, the publication of these (S)SCI papers can provide a direct promotion track to full professor, the highest academic title in China, regardless of teaching and other professional practices (Shu et al., 2020). In some cases, the excessive emphasis on research publication makes other efforts, especially efforts in T&I practice, meaningless. That is why, though T&I teachers perceived great enthusiasm and excitement toward T&I practice, when talking about competing time and energy, the first thing they gave up was T&I practice.

As a result, the relationship between the roles of researcher and practitioner, a non-overlapping yet overshadowing one, is demonstrated visually in Figure 4 by the TITRI framework as the researcher role eclipses the practitioner role, rather than overlapping, as Figure 3, because the former overshadows rather than merges or integrates with the latter.

#### Overlapping and overshadowing role identities

Rather than a wide divide between the researcher and practitioner roles as described by T&I teachers, they sense both the direct and indirect links between the trainer/educator and practitioner roles as they aim to utilize their practice experience to be a “role model for students.” More specifically, the T&I teachers’ perceptions of combining training classes with authentic translation materials, as well as their desire to help the community during COVID-19, all act as components in their T&I practitioner role identity systems, which are aligned to inform the perception and goals of the T&I trainer/educator role identity system. The connection between the goals of providing a role model for students and the accompanying action possibilities and self-perception of value fulfillment leads to T&I teachers seeing the experience as rewarding and meaningful. This positive emotion reinforced the integration of the two roles.

However, the trainer/educator role identity has once again taken precedence over the practitioner one. T&I teachers are required to teach a certain number of courses that are directly relevant to their job obligations as full-time university teachers, but they are not required by institutions to participate in T&I practice. To be more precise, no matter how seasoned a practitioner is or how well-received by their employers, a T&I teacher cannot be promoted to a higher academic title without high-impact publications and satisfying the teaching duties. The teacher/faculty appraisal mechanism and the promotion system thus have given rise to an enhancement of the T&I trainer/educator role but a diminishment of the T&I practitioner role.

Figure 5 depicts both the integration and secondary positions of the T&I practitioner role in light of Figures 3, 4 presented earlier. It differs from Figure 3 in that it emphasizes the practitioner’s secondary position, which is not discovered between trainer/educator and researcher role identities. It differs from Figure 4 in that it stresses the overlapping connection between trainer/educator and practitioner roles, which has not been identified between researcher and practitioner roles.

#### An overall translation and interpreting teacher role identity framework

After analyzing the dynamic interaction between two role identities (Figures 3–5), we placed all the roles into a T&I teacher role identity system to make it a wholesome framework (Figure 6). When placing all the roles into a unified framework, the complexity is even greater, because each role identity is part of the larger system of T&I teacher role identity. Whether the three sub-roles support one another or compete against one another, they are nevertheless merged in the main role of the T&I teacher.

As the data reveal, participants are enthusiastic and satisfied with their major role identity as a T&I teacher, shedding light on how T&I teachers perceive disintegration between three sub-role components in their DSMRI system. First, due to the high loyalty to the main role, teacher in higher education, we can rightly anticipate that the participants would be motivated to make use of all available recourses, promoting themselves to further adapt to the requirements of the main role. Furthermore, since they never want to change jobs, we can expect that T&I teachers’ beliefs within three sub-roles will be linked with professional goals in staying in the position even if they face temporary difficulties and setbacks. Finally, because of their long-standing attachment to the teaching role, they are unlikely to set goals and perceive action possibilities that are not conducive to the development of their main role identity.

An overall TITRI framework (Figure 6) thus puts three sub-roles in an enclosed main role identity system. It stresses the need of understanding how sub-roles interact and how they relate to the main role, while also placing them in a coherent framework and allowing generalization among the roles.

Together, the TITRI framework articulates the interplay of role identities and the consequences of higher education on the role identity formation of T&I teachers. It may be used to frame future research in a particular role, understand T&I teacher agency and autonomy (Huang, 2021; Huang et al., 2021; Huang and Yip, 2021), or gain new insights into how T&I teacher role identities are formed and what they provide to higher education. The overshadowing interaction highlights the institutional impacts on the construction of teacher role identity. To understand why the T&I practitioner identity is overshadowed by the other two role identities, one should not leave out the context of higher education, where T&I practice is a secondary paid activity and a university job is the principal source of income (Torres-Simón and Pym, 2016). To explain why researcher identity is more important for T&I teachers, one must also consider the background of the expansion and internationalization of higher education in China, which places a greater emphasis on research achievement than teaching and service excellence (Shu et al., 2020).

## Conclusion

Based on the DSMRI, the TITRI framework aims to provide a broad overview of how role identities interact with one another and ultimately merge as a T&I teacher. The framework first clearly articulates the interaction between trainer/educator, researcher, and practitioner role identities and how they relate to T&I teacher identity development. Despite the fact that previous research (e.g., Kelly, 2008; Orlando, 2016) has placed T&I teachers at the crossroads of training, research, and practice and has identified the interaction and correlation between them as an asset rather than a burden for T&I teachers, the connections between these different roles have yet to be clearly established. The TITRI framework thus fills the gap by providing a clear and visual representation of the links. The articulation enables teachers with greater preparation, more accessible opportunities, and a more efficient means of comprehending their role interaction (Dornsife, 1992). It is also hoped that this study adds to the exploration of how pedagogical, theoretical, and professional components might be integrated (Orlando, 2016) and to the search for better T&I teacher development support (Kelly, 2008; Wu et al., 2019a).

The study is not without its limitations. First and foremost, the study only focuses on T&I teachers’ engagement in training/educating, research, and practice. We acknowledge that T&I teachers may assume additional academic and administrative duties (Guberman and Mcdossi, 2019), such as department head, and that these responsibilities may impact their trainer/educator, researcher, and practitioner role identities. Nevertheless, the study narrows to focus on the three most assumed sub-roles by T&I teachers based on the knowledge structure of T&I teachers (Li and Zhang, 2011) and the main role of a university teacher in China. In addition, the research cannot speak to how the social identities of T&I instructors may impact participants’ beliefs, perceptions, self-definitions, and action possibilities. The present study fully recognizes that the personal factors and social aspects are important considerations for their professional development, such as age, gender, and sexuality (Vavrus, 2009), and parenthood (Henry, 2021). Though there is still more work to be done to examine T&I teacher role identity, the TITRI framework itself is effective in stimulating discussion and reflection about the professional role identity development of T&I teachers.

## Author information

**Bacui Chen (first author):** BC is a doctoral candidate in the Department of Education Studies at Hong Kong Baptist University. She is also a lecturer of translation and interpreting in Lingnan Normal University in Guangdong Province, China. Her research interests include translation and interpreting training, and teacher identity/agency/autonomy. She has published several articles in Chinese journals. She has translated over 10 best-selling books from English to Chinese, including *Pacific Rim series*.

**Jing Huang (second and corresponding author):** JH obtained his Ph.D. degree in Applied Linguistics from the University of Hong Kong. He has been working as a language teacher educator for more than 20 years in mainland China, Singapore, and Hong Kong. His research is in autonomy/identity/agency in second language education, TESOL teacher education, educational ethnography, and metacognition in language learning. He has published extensively in these areas in reputable journals including *System; Language Teaching Research*; *The Modern Language Journal*; *TESOL Quarterly; Language, Culture and Curriculum; Asia Pacific Journal of Education; Frontiers in Psychology; Sustainability; SAGE Open; Educational Action Research; Teaching Education; etc.* Google Scholar profile: https://scholar.google.com/citations?user=jJrsIUkAAAAJ&hl=en&oi=ao.

## Data availability statement

The original contributions presented in this study are included in the article/supplementary material, further inquiries can be directed to the corresponding author.

## Ethics statement

The studies involving human participants were reviewed and approved by Hong Kong Baptist University Committee on the Use of Human and Animal Subjects in Teaching and Research (HASC). The patients/participants provided their written informed consent to participate in this study.

## Author contributions

BC led the research project and contributed to the research design, data collection, data analysis, article drafting, and revising. JH analyzed the data, reviewed, and revised the manuscript. Both authors have approved the submission.
